# Suggestion of a simpler and faster influenza-like illness surveillance system using 2014–2018 claims data in Korea

**DOI:** 10.1038/s41598-021-90511-0

**Published:** 2021-05-27

**Authors:** HeeKyoung Choi, Won Suk Choi, Euna Han

**Affiliations:** 1 College of Pharmacy, Yonsei Institute of Pharmaceutical Research, Yonsei University, 162-1 Songdo-dong, Yeonsu-gu, Incheon, Seoul, Republic of Korea; 2 Division of Infectious Diseases, Department of Internal Medicine, National Health Insurance Service Ilsan Hospital, Ilsan, Republic of Korea; 3 Division of Infectious Diseases, Department of Internal Medicine, Ansan Hospital, Korea University College of Medicine, Ansan, Republic of Korea

**Keywords:** Public health, Infectious diseases, Epidemiology

## Abstract

Influenza is an important public health concern. We propose a new real-time influenza-like illness (ILI) surveillance system that utilizes a nationwide prospective drug utilization monitoring in Korea. We defined ILI-related claims as outpatient claims that contain both antipyretic and antitussive agents and calculated the weekly rate of ILI-related claims, which was compared to weekly ILI rates from clinical sentinel surveillance data during 2014–2018. We performed a cross-correlation analysis using Pearson’s correlation, time-series analysis to explore actual correlations after removing any dubious correlations due to underlying non-stationarity in both data sets. We used the moving epidemic method (MEM) to estimate an absolute threshold to designate potential influenza epidemics for the weeks with incidence rates above the threshold. We observed a strong correlation between the two surveillance systems each season. The absolute thresholds for the 4-years were 84.64 and 86.19 cases per 1000claims for claims data and 12.27 and 16.82 per 1000 patients for sentinel data. The epidemic patterns were more similar in the 2016–2017 and 2017–2018 seasons than the 2014–2015 and 2015–2016 seasons. ILI claims data can be loaded to a drug utilization review system in Korea to make an influenza surveillance system.

## Introduction

Influenza, which can cause epidemics and pandemics through antigenic shift, and localized outbreaks through antigenic drift^1^, is one of the most important infectious diseases in public health^2^. Therefore, we need a national surveillance system to monitor and respond to an influenza epidemic. In Korea, such a system is currently based on a clinical sentinel surveillance system, a laboratory sentinel surveillance system, and a hospitalization and mortality monitoring system, all of which use information collected from a restricted number of selected outpatient clinics^3,4^. This traditional influenza surveillance system is considered the gold standard for relevant studies and public interventions^5–7^. The clinical sentinel surveillance system reports the proportion of influenza-like illness (ILI) visits among weekly outpatients through the voluntary participation of 200 local clinics nationwide. This system has been well used in Korea for a long time^8^. However, due to the small number of participating clinics in each region, calculating the ILI rate by region or age is limited. In addition, as pediatric clinics account for half of the 200 surveillance clinics^8^, it is possible that the entire epidemiology cannot be sufficiently reflected. As the surveillance system relies on voluntary reporting, there is a concern of under-reporting. Also, the degree of under-reporting may differ depending on the characteristics of the clinic because case reporting can be affected by the patient volume and the physician’s compliance^9^.

Patient visit, data submission, analysis of weekly ILI rates and public announcement take one to two weeks^5^, and such a time lag from actual peak to alarm is inevitable. The external validity of these reports has not been determined. Such caveats are widely recognized globally, and an alternative system using prescription drug sales^10,11^, emergency department use^12^, or school absenteeism data^13^ has been proposed. More recently, research has attempted to predict the incidence of ILI in real-time using novel data from Google or other social media^14,15^, Wikipedia logs^16,17^, Twitter^18^, or a combination of multiple data sources^19^. These methods are limited in their generalization to other regions^20^, and may still have instabilities, such as overestimation^21^. Koreans need a timely and valid surveillance system to complement the sentinel surveillance system^5^.

We sought to develop a simpler and faster surveillance system using real-time prospective drug utilization system in Korea. There is only one public health insurer in South Korea, and all Koreans and legally-residing foreigners are mandatory beneficiaries and all medical institutions (including pharmacies) are compulsory providers. Therefore, the single-payer National Health Insurance (NHI) system of Korea allows national and regional level research of healthcare service use for the entire population or representative samples in Korea. Given the comprehensive coverage of insurance under the NHI, the access barrier to healthcare service use is relatively low and thus sample selection is less of a concern. The Health Insurance Review and Assessment Service (HIRA) in Korea operates a drug utilization review (DUR) system that monitors the prescribing and dispensing of drugs nation-wide and in real-time. Therefore, by monitoring the rate of drug prescriptions related to ILI, we can capture the ILI status by country-wide and sub-regional in real time. To prove this, we defined ILI-related prescription claims, calculated the rate of the ILI-related claims among the total claims, and assessed its association with the existing clinical sentinel surveillance system.

## Results

**Figure 1:**
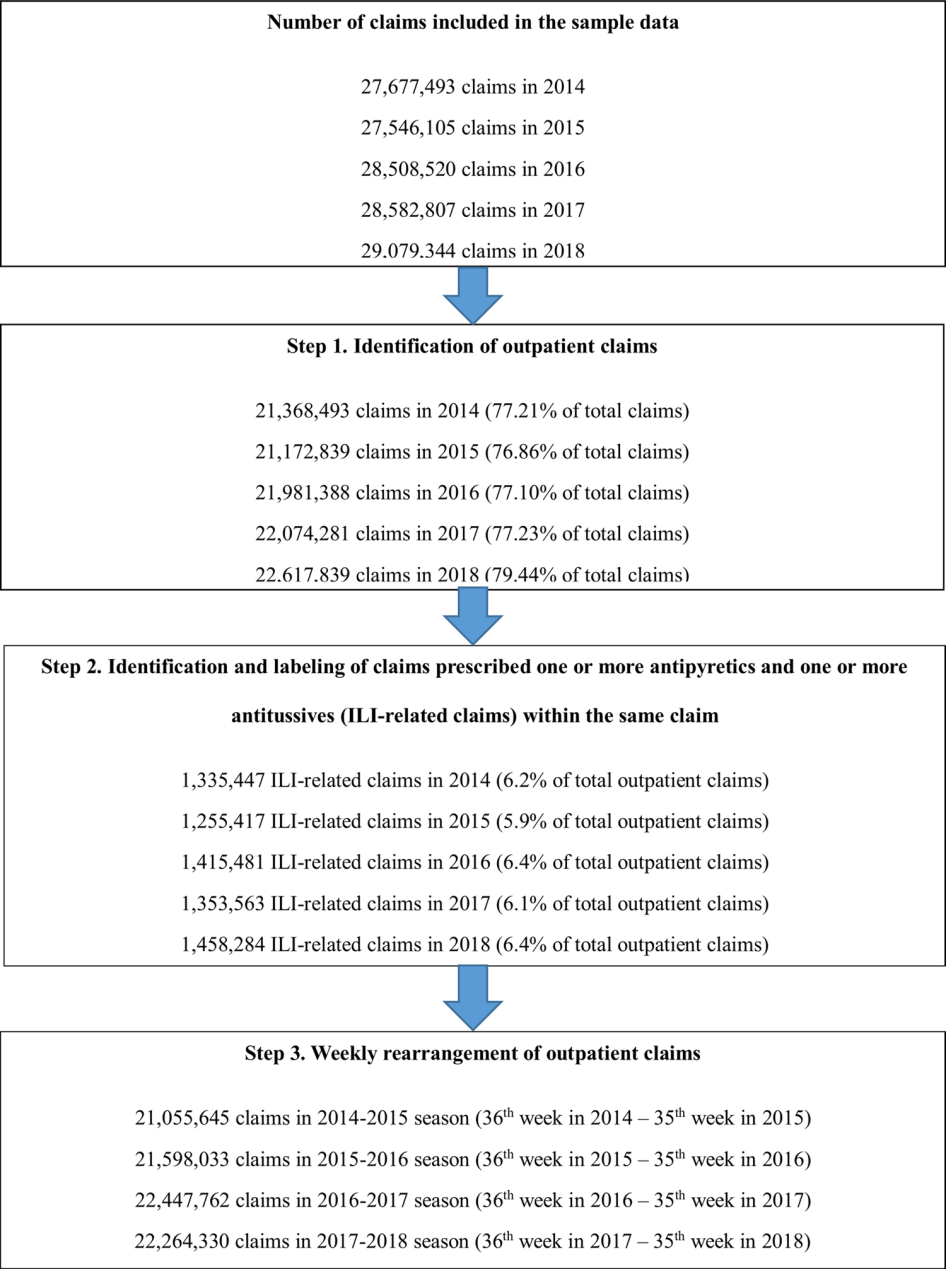
Defining ILI-related claims.

We collected a total of 208 weeks of ILI data from Korea Centers for Disease Control and Prevention (KCDC) and outpatient claims data from the NHI system from 2014 to 2018. The epidemic peak in the ILI data was observed in the 8th week (45.5 ILI visits per 1000 patients) in the 2014–2015 season, the 7th week (53.8 ILI visits per 1000 patients) in the 2015–2016 season, the 52nd week (86.2 ILI visits per 1000 patients) in the 2016–2017 season, and the 1st week (72.1 ILI visits per 1000 patients) in the 2017–2018 season. There was a total of 109,214,840 NHI outpatient claims during the 2014–2018 period. The proportions of ILI-related claims to the total outpatient claims were 6.2%, 5.9%, 6.4%, 6.1%, and 6.4% in 2014, 2015, 2016, 2017, and 2018, respectively. Those claims were arranged weekly as the 36th week of the previous year to the 35th week of the years 2014–2015, 2015–2016, 2016–2017, and 2017–2018. Approximately 21–22 million claims were included in each season. Similar to the sentinel surveillance data, the epidemic peak in the ILI data was observed in the 8th week in the 2014–2015 season, the 7th week in the 2015–2016 season, the 52nd week in the 2016–2017 season, and the 1st week in the 2017–2018 season (Fig. 1).

**Figure 2:**
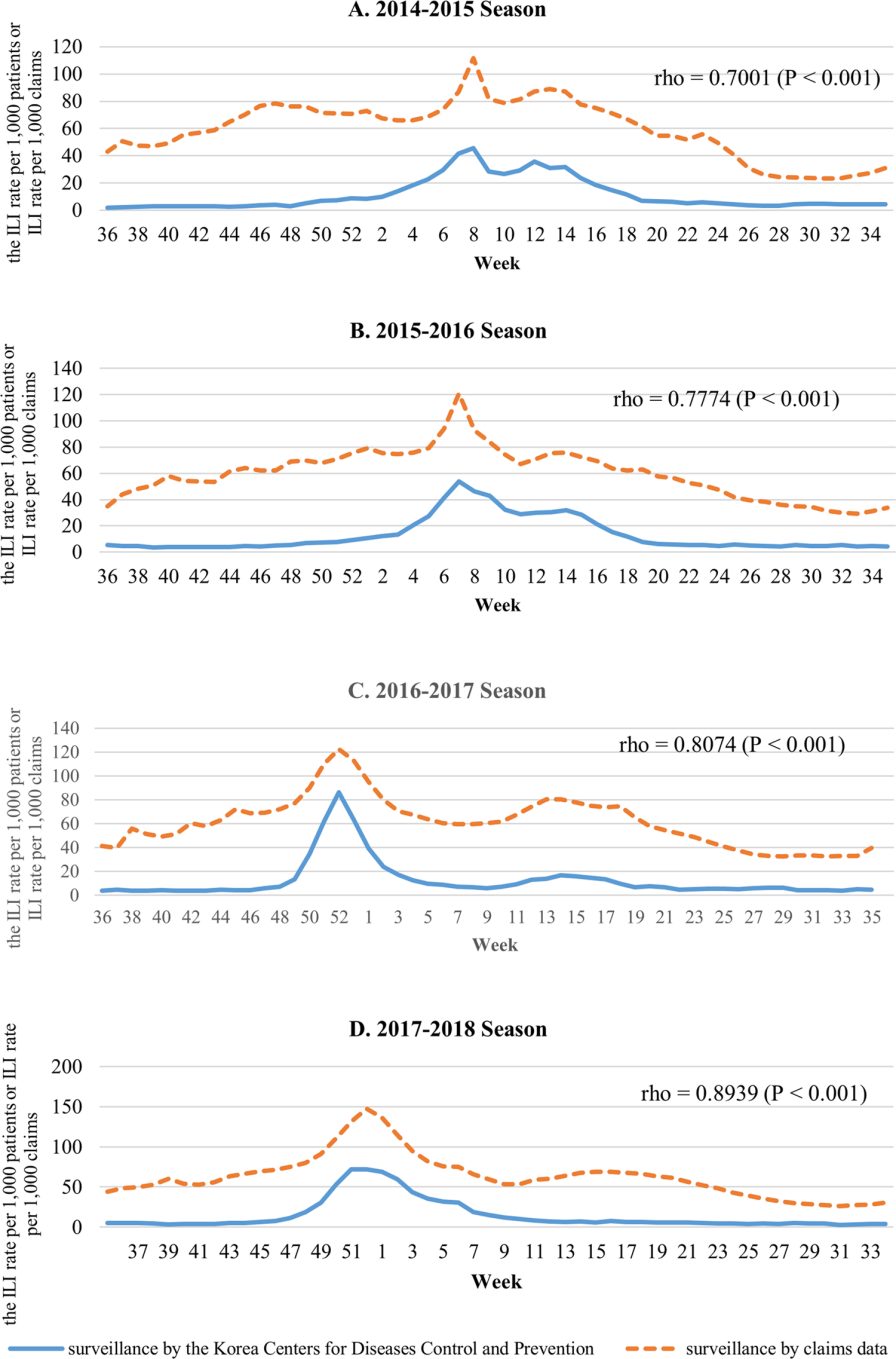
**(A)** Comparison of the ILI-related claims rate and clinical sentinel surveillance report, 2014–2015 season. **(B)** Comparison of the ILI-related claims rate and clinical sentinel surveillance report, 2015–2016 season. **(C)** Comparison of the ILI-related claims rate and clinical sentinel surveillance report, 2016–2017 season. **(D)** Comparison of the ILI-related claims rate and clinical sentinel surveillance report, 2017–2018 season.

Figure 2 shows the relationship between the ILI-related claims rate and the reported ILI rate from the KCDC data. The two surveillance systems show similar trends. A strong correlation was observed over each season (2014–2015 season, rho = 0.7001, P < 0.001; 2015–2016 season, rho = 0.7774, P < 0.001; 2016–2017 season, rho = 0.8074, P < 0.001; and 2017–2018 season, rho = 0.8939, P < 0.001).

When comparing the ILI-related claims rate and the KCDC surveillance data rate by age group, the correlation coefficient was the highest in the 50–64-year-old age group for all three seasons (rho = 0.7735 for the 2014–2016 season, rho = 0.7455 for the 2014–2015 season, and rho = 0.8283 for the 2015–2016 season). For the 50–64-year-old age group, the incidence rates peaked at week 8 in the 2014–2015 season and week 7 in the 2015–2016 season (42.4 cases per 1000 patients in the 2014–2015 season and 33.1 cases per 1000 patients in the 2015–2016 season for KCDC data, and 106.9 claims per 1000 claims in the 2014–2105 season and 118 claims per 1000 claims in the 2015–2016 season for NHI claims data). The correlations were statistically significant for all other age groups, with the correlation coefficients ranging from 0.5529 to 0.7732 across seasons (Table 1).

**Table 1:** Comparison of the weekly ILI-related claims rate and clinical sentinel surveillance report, 2014–2016.

	ILI claims	Total claims	Number of observations (week)	Rho	P-value*
**2014–2016 season**					
0–6 years of age	244,804	4,857,228	104	0.6350	** < 0.001**
7–18 years of age	334,639	3,287,888	104	0.6865	** < 0.001**
19–49 years of age	918,294	12,121,075	104	0.7078	** < 0.001**
50–64 years of age	582,828	10,748,959	104	0.7735	** < 0.001**
65 +	493,298	11,638,528	104	0.7208	** < 0.001**
All	2,573,863	42,653,678	104	0.7328	** < 0.001**
**2014–2015 season**					
0–6 years of age	116,658	2,387,055	52	0.5529	** < 0.001**
7–18 years of age	168,546	1,638,138	52	0.6755	** < 0.001**
19–49 years of age	449,593	6,027,427	52	0.6537	** < 0.001**
50–64 years of age	289,251	5,272,972	52	0.7455	** < 0.001**
65 +	250,284	5,730,053	52	0.7234	** < 0.001**
All	1,274,332	21,055,645	52	0.7001	** < 0.001**
**2015–2016 season**					
0–6 years of age	128,146	2,470,173	52	0.7057	** < 0.001**
7–18 years of age	166,093	1,649,750	52	0.7376	** < 0.001**
19–49 years of age	468,701	6,093,648	52	0.7647	** < 0.001**
50–64 years of age	293,577	5,475,987	52	0.8283	** < 0.001**
65 +	243,014	5,908,475	52	0.7732	** < 0.001**
All	1,299,531	21,598,033	52	0.7774	** < 0.001**

Since HIRA's age group has been provided differently from KCDC since 2017, only the 2014–2016 data set was used for analysis by age group.

*P < 0.05 is considered statistically significant (indicated by bold text).

**Figure 3:**
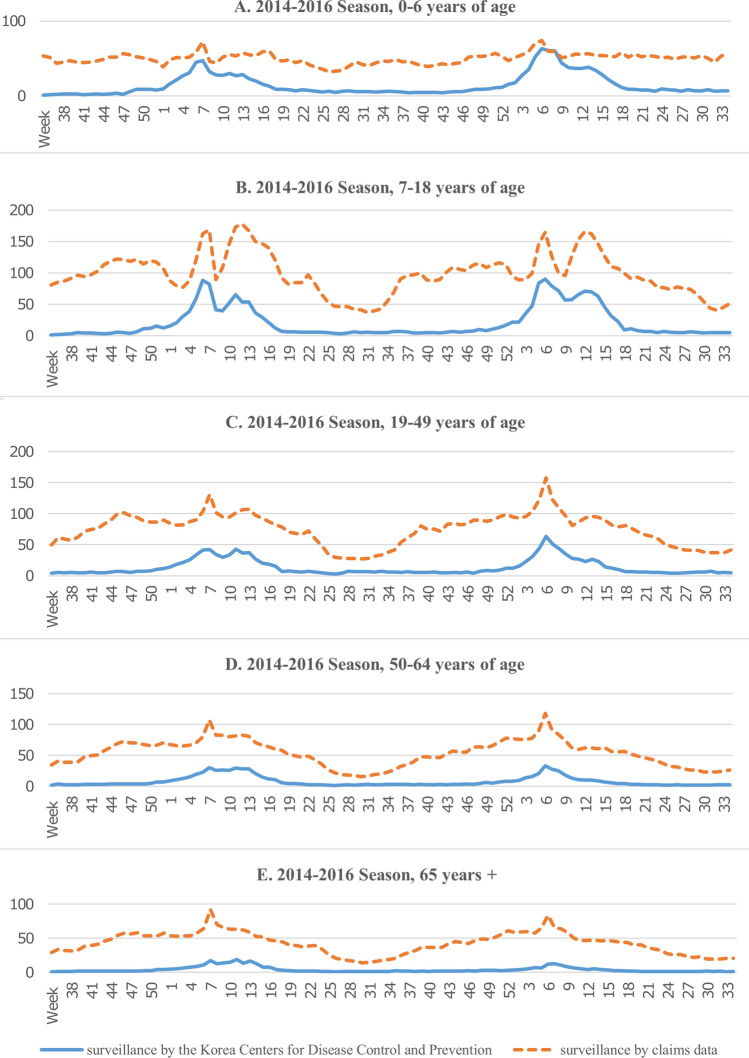
Comparison of the ILI-related claims rate and clinical sentinel surveillance report by age group, 2014–2016.

Figure 3 shows the ILI trends according to age group during the 2014–2016 seasons. In most weeks and age groups, the ILI-related claims rates were higher than the KCDC reported ILI rates. In individuals under six years of age, there was a more pronounced fluctuation in the KCDC rate than the ILI-related claims rate. Conversely, in those aged 65 years and older, the ILI-related claims rate showed significant fluctuation compared to the KCDC rates.

**Figure 4:**
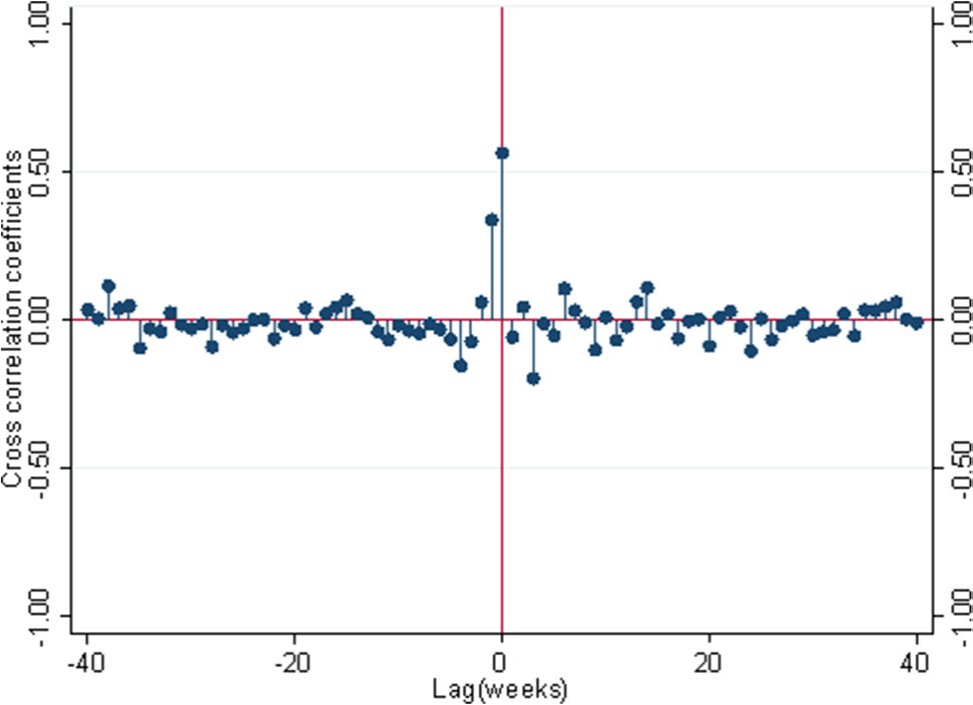
Cross-correlation between the KCDC sentinel ILI rates and National Health Insurance claims ILI rates.

We examined the partial autocorrelation function for sentinel and claims surveillance data, respectively, which showed a statistically significant autocorrelation at the 5% level for a lag of one week for the sentinel data and a lag of two weeks for the claims data (results not shown). The augmented Dickey-Fuller test for the null hypothesis of the unit root process (i.e., non-stationarity) was not rejected at the 5% level for either data, i.e., there was no statistically significant persistent time series, and differencing was not applied. We applied the autoregressive function for respective lags for each dataset and examined the cross-correlation coefficients between the two datasets. The residuals for the correlations were approximately normally distributed (results not shown). Figure 4 shows the cross-correlations between the residuals of the first-order (sentinel data) or the second-order (claims data) autoregressive function of each data, representing a gradual decrease in the correlation coefficients for lags greater than zero. These results indicate that the claims data neither lead nor lag the sentinel data.

**Figure 5:**
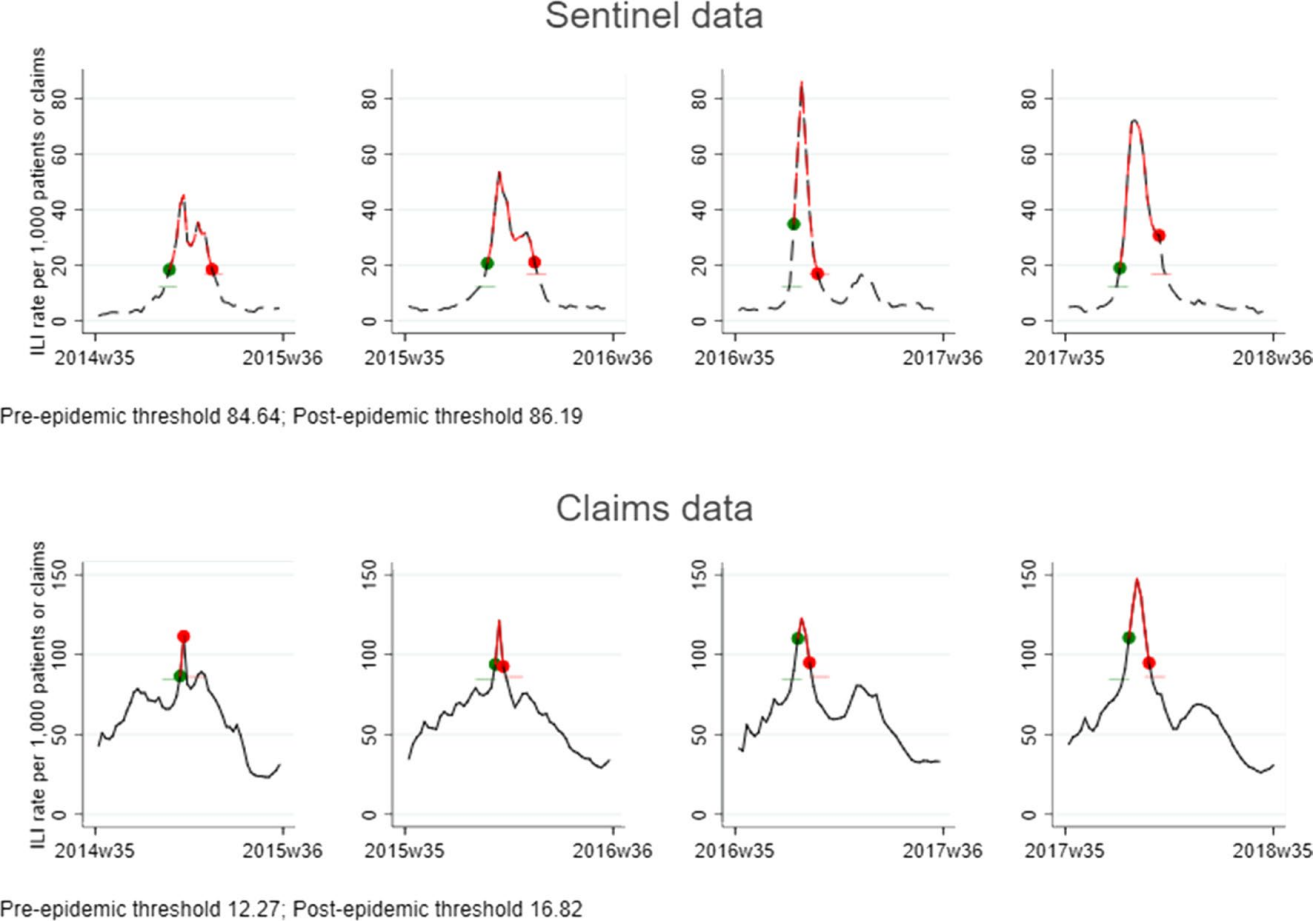
Epidemic thresholds for the surveillance of ILI rates from the KCDC sentinel data and National Health Insurance claims data.

Figure 5 and Supplementary Fig. 1 show the MEM analysis results. The absolute thresholds for the four-year surveillance period (2014–2018) were 84.64 and 86.19 claims per 1,000 claims for the claims data and 12.27 and 16.82 per 1000 patients for the sentinel data (Supplementary Fig. 1). Both the claims and sentinel data surpassed the respective epidemic threshold in each of the four seasons. The epidemic was relatively longer in the sentinel data than the claims data, and the epidemic peaked in the claims data one to two weeks later than in the sentinel data. The epidemic pattern showed greater similarity in terms of the peak during the epidemic period in the 2016–2017 and 2017–2018 seasons than the 2014–2015 and 2015–2016 seasons (Fig. 5).

## Discussion

The influenza surveillance system of Korea is operated by the KCDC and consists of the three systems; clinical sentinel surveillance, laboratory sentinel surveillance, and an influenza hospitalization and mortality surveillance system^3,4^. Among them, the clinical sentinel surveillance system began operation on a pilot basis in 1997 with more than 70 private medical institutions. In 2000, the system was expanded to the Korea Influenza Surveillance Scheme, which consists of a clinical and a laboratory monitoring system involving public health centers and private medical institutions. In 2008, the public health center's zero reporting rate was high, even during the influenza epidemic, so the public health center was replaced with a private medical institution to secure more reliable data. In 2009, the number of monitoring agencies was expanded, but there was still a limit to the number of zero reporting sites even during an epidemic period. During expert meetings held in 2013, 200 clinical surveillance institutions were designated as active participants, 36 of which were invited to participate in a laboratory sentinel surveillance system. Currently, the clinical sentinel surveillance involves the selection of 200 outpatient clinics, designated by a medical association, including 100 pediatric clinics and 100 internal or family medicine clinics with specialties in internal medicine, pediatrics, and family medicine.^8^ Site selection is based on geographical distribution and population characteristics (Supplementary Table 3)^22^. The number of ILI patients and the total number of outpatients should be reported weekly from April to November of the current year and daily from December to April of the following year. After collecting each clinic’s data, KCDC releases the proportion of ILI visits per 1000 patients per week each week.

However, one disadvantage to such a surveillance system is that a one to two-week reporting lag is inevitable. Considering the potential mismatch of influenza vaccines and circulating virus strains and the limited capacity to prevent an influenza epidemic in advance^23^, it is essential to control an epidemic promptly^5^. Such a sentinel alert system is also time and labor-intensive: a network of 400 outpatient providers across Korea submit ILI counts, which are then summarized by the KCDC^8^. Also, reports may not always reflect the trends across the country^5,24^, as participation is voluntary^8^ and results are from conveniently selected sample clinics^8^. This can lead to under-reporting, especially early in the season, when both doctors and the general population are unaware of an influenza outbreak. Several weeks may pass between the actual onset of a seasonal epidemic and the official alert. As reporting can be affected by the patient volume and the physician’s compliance^9^, the amount of under-reporting varies from clinic to clinic.

Therefore, new surveillance systems attempt to overcome these shortcomings and detect early influenza signals^14,15,19,25–30^. Google Flu Trends was a web service that predicted an influenza epidemic based on 45 queries^14^. Although initial reports had a 97% accuracy compared to the United States Center for Disease Control data^31^, subsequent reports revealed Google Flu Trends to be inaccurate^21^. The errors in Google Flu Trends may have been due to media-stoked panic and the fact that people making flu-related searches may not have symptoms, but were researching other disease symptoms similar to influenza^21^. Other social media platforms, such as Twitter, have made efforts to remove unrelated messages to improve programme accuracy^18^. Regardless, searches on Google, Wikipedia, or social media are likely to vary from country to country^30^. The coverage even within a country varies, as rural areas with older populations are likely to have low access to social media^18^ and are thus systematically underrepresented. External validation of algorithms using this data is questionable.

ILI does not have a diagnostic code that is adequately responsive. By contrast, drug prescription data are reliable because they reflect the doctor’s judgment regarding the patient’s condition. Therefore, claims with antiviral agents for influenza can be used for surveillance, which is likely to detect confirmed influenza cases. Claims data, including prescription records, are valid as the patient’s subjective symptoms are confirmed by the prescribing physician. A surveillance system based on claims data can predict influenza epidemics more efficiently and reliably than internet search engines or a self-report system, without any additional data collection. The national health insurance system in South Korea is particularly useful for complementary influenza surveillance given that the claims data represents healthcare service use for the entire population, and most of the services are reimbursed.

There may be a time lag between the actual practice and the reimbursement claim. Therefore, we propose applying this study’s concepts to the nationwide Drug Utilization Review (DUR) system in South Korea. DUR is operated by the HIRA, which has all of the reimbursement claims data for review and assessment. The DUR system detects potential side effects and unsafe use at the time of prescribing and dispensing the medications, providing patient records of drug use and real-time alerts^32^. In this system, a physician’s prescription is cross-checked by the HIRA using a specialized database that can warn doctors via their computer screen in real-time (Supplementary Fig. 2). The doctor may then change the prescription or note the reason they are keeping it as is. The final decision will be sent to HIRA. Pharmacists will follow the same process. A warning message on the DUR system prompts the pharmacist to follow-up with the doctor before dispensing the medication. Final dispensing details are sent to the HIRA. This process is completed within 0.5 s. All providers in Korea have access to the DUR system and send prescription and dispensing details to the HIRA. Therefore, ILI surveillance can be performed in real-time by adding an indicator for ILI-related claims in the DUR system.

The proposed alert system is similar to a real-time alert system for influenza epidemics in other countries, including Japan and Taiwan^5,24^. Taiwan’s system proactively sends an alert to subscribers via a mobile application or computerized physician order entry when one of the three surveillance systems, based on the sentinel reports, insurance claims, or the electronic medical records of selected hospitals, presents incidence values over the epidemic threshold^5^. However, planting the surveillance system in the prospective DUR is a more novel and efficient manner of capturing and controlling the emerging surveillance alert given that it is based on the compulsory participation of all pharmacies and clinicians in a nation. This ensures that all healthcare providers are subscribers and the government is already part of the system.

There are several limitations in this study. First, we did not compare ILI claims rates with laboratory-confirmed influenza cases. The ILI rates do not represent true cases of influenza. ILI has a low sensitivity (30–70%) for predicting laboratory confirmed influenza^33,34^. Patients who were diagnosed with laboratory confirmed influenza are prescribed antiviral agents, but not everyone is prescribed antiviral agents. Moreover, some antiviral agents are not covered by insurance. Therefore, all confirmed influenza cases are not detected by claims data alone, and laboratory data is required to ensure that influenza-specific policies are initiated. We also acknowledge that monitoring ILI-related prescription claims does not provide information about the virus or disease severity and cannot replace the entire influenza surveillance system. The system suggested in this study cannot replace all of the traditional influenza surveillance systems, and the need for laboratory or hospitalization and mortality surveillance still exists. Regardless, this may complement the traditional clinical sentinel surveillance to provide quicker and easier data collection and analysis^5^. Second, national health insurance in Korea is a single-payer program, and has an electronic prescription monitoring system that streamline real-time data collection and reporting. Therefore, implementation of this scheme is limited in other countries with other health insurance systems. Third, physicians may avoid prescribing antitussive agents despite the patient’s symptoms. This trend is expected to occur in young children, because it is recommended to use antitussive agents more carefully in younger children due to concerns about side effects^35^. In conclusion, the weekly fraction of outpatient claims having both antipyretic and antitussive agents among the total claims were similar to the existing sentinel ILI surveillance system. This suggests that it is possible to integrate a new, real-time influenza surveillance system to the existing system for efficient and timely surveillance.

## Methods

### Data source

We used the Health Insurance Review and Assessment Service-National Patient Samples (HIRA-NPS), 2014–2018, to identify ILI-related insurance claims. The HIRA-NPS is a stratified random sample of 3% of the Korean population that includes approximately 1.4 million individuals. Because the HIRA-NPS extracts data from the National Health Insurance System (NHIS), it only includes data on claims that are reimbursed by the NHIS^36^.

National weekly ILI rates were pulled from public data provided by the Korea Centers for Diseases Control and Prevention (KCDC)^37^. The selected sites for the clinical sentinel surveillance system report the number of ILI patients and the number of outpatients. Based on this report, KCDC releases the weekly incidence of ILI per 1000 patients.

To reinforce personal information protection, HIRA has revised the principle of providing data for research from 2017. They offered only the age group instead of the exact age, and the age groups of KCDC and HIRA were different. Therefore, only data prior to 2017 was used for comparison by age group.

### Operational definition of ILI

ILI is defined as an acute respiratory illness with a measured temperature of 38.0 °C or higher and a cough with onset within the last 10 days^38^. For sentinel surveillance, ILI is defined as the sudden onset of fever (> 38.0 °C) with cough or sore throat^39^. The ILI rate, calculated using the total number of weekly outpatient patients as the denominator and the number of weekly patients with ILI as the numerator, is reported as the number of ILI patients per 1000 outpatients per week.

We defined an influenza-like illness (ILI)-related claim as an outpatient claim that contains both antipyretic and antitussive agents (Supplementary Table 1, 2). The ILI-related claims rate was defined as the proportion of ILI-related claims of the entire outpatient claims for a given period. We used the following process to define an ILI-related claim (Fig. 1): (1) we pulled outpatient claims; (2) we identified those outpatient claims with both antipyretic and antitussive agents (ILI-related claims, hereafter); (3) we reorganized claims weekly, like the clinical sentinel surveillance data issued by the KCDC; and (4) the ILI-related claims rate was calculated as the weekly number of ILI-related claims per 1000 outpatient claims per week.

### Statistical analysis

We first performed a cross-correlation analysis using Pearson’s correlation to compare ILI-related claims rates and weekly ILI rates from the KCDC’s clinical sentinel surveillance data. We then used a time-series analysis to examine the autocorrelation in both surveillance data to explore actual correlations after removing any dubious correlations due to underlying non-stationarity in both data sets. We performed the partial autocorrelation function for all lags and the augmented Dickey-Fuller test for the null hypothesis of the unit root process (i.e., non-stationarity)^40,41^. We then generated cross-correlation coefficients for residuals after controlling for any autocorrelation properties. Lastly, we used the moving epidemic method (MEM) to estimate an absolute threshold from historical influenza incidence data and to designate potential influenza epidemics for the weeks with incidence rates above the threshold^42,43^. We estimated the absolute threshold for the sentinel data and insurance claims data, respectively, using the MEM, and compared the epidemic period and whether the peak in the epidemic period is visually overlapped for each data set. Data were analyzed weekly over 12 months for the entire study duration and separately by each surveillance period starting from the 36th week of a calendar year to the 35th week of the following calendar year.

Data analysis was performed using SAS software, version 9.4 (Cary, NC), Stata software version 14 (StataCorp, College Station, TX, USA, https://www.stata.com/stata14/), and R 3.4.2 (CRAN) using the MEM library version 2.11^44^.

### Ethical statement

This study was approved by the Institutional Review Board (IRB) of Yonsei University (approval number: 201905-h-1535-01) and Korea University Ansan Hospital (approval number: 2019AS0107).

## Supplementary Information

Supplementary Information.
