# Supplementary material for: Suggestion of a simpler and faster influenza-like illness surveillance system using 2014–2018 claims data in Korea
Source: Sci Rep. 2021 May 27;11:11243. doi: 10.1038/s41598-021-90511-0 (PMC8159991; doi:10.1038/s41598-021-90511-0)

Suggestion of a simpler and faster influenza-like illness surveillance system: verification using 2014- 2018 claims data in Korea

Authors: HeeKyoung Choi, Won Suk Choi, Euna Han*

Author affiliations:

College of Pharmacy, Yonsei Institute of Pharmaceutical Research, Yonsei University, Seoul, Republic of Korea (HeeKyoung Choi, Euna Han)

Division of Infectious Diseases, Department of Internal Medicine, Korea University College of Medicine, Ansan Hospital, Ansan, Republic of Korea (HeeKyoung Choi, Won Suk Choi)

**HeeKyoung Choi and Won Suk Choi contributed equally as a first author.*

*** Corresponding Author:**

Euna Han, PhD (https://orcid.org/0000-0003-2656-7059)

College of Pharmacy, Yonsei Institute of Pharmaceutical Research

Yonsei University

162-1 Songdo-dong, Yeonsu-gu, Incheon, Korea

Phone: +82-32-749-4511; Fax: +82-32-749-4105

E-mail: eunahan@yonsei.ac.kr

Supplementary Table 1. ATC codes of antipyretics

ATC names	Drug code	ATC code
Butylpyrazolidines	221301ACH	M01AA
Acetic acid derivatives and related substances	166101ACH	M01AB
Indomethacin	174803ACH	M01AB01
Sulindac	233201ATB	M01AB02
	233203ATB	M01AB02
Tolmetin	501201ATB	M01AB03
	501202ACH	M01AB03
	501203ATB	M01AB03
Diclofenac	143402ATB	M01AB05
	143504ATB	M01AB05
	143506ATB	M01AB05
	143534BIJ	M01AB05
	143535BIJ	M01AB05
	143630BIJ	M01AB05
	143631BIJ	M01AB05
	143401ATB	M01AB05
	143402ATB	M01AB05
	143404BIJ	M01AB05
	143501ACR	M01AB05
	143502BIJ	M01AB05
	143504ATE	M01AB05
	143534BIJ	M01AB05
	143535BIJ	M01AB05
	143601BIJ	M01AB05
	143630BIJ	M01AB05
	143631BIJ	M01AB05
etodolac	156601ATB	M01AB08
	156602ACH	M01AB08
	156602ATB	M01AB08
	156603ATB	M01AB08
	156603ATR	M01AB08
	156604ATR	M01AB08
lonazolac	185001ATB	M01AB09
proglumetacin	218602ATB	M01AB14

	218601ACH	M01AB14
	218603ACH	M01AB14
ketorolac	180001ATB	M01AB15
	180033BIJ	M01AB15
	180001ATB	M01AB15
	180002BIJ	M01AB15
	180005BIJ	M01AB15
	180008BIJ	M01AB15
	180035BIJ	M01AB15
aceclofenac	100901ACH	M01AB16
	100901ACS	M01AB16
	100901ATB	M01AB16
	100903ATR	M01AB16
	100901ACH	M01AB16
	100901ATB	M01AB16
	100903ATR	M01AB16
diclofenac, combinations	251900ATB	M01AB55
Oxicams	133801ATB	M01AC
piroxicam	214031BIJ	M01AC01
	214202ATB	M01AC01
	214202ATB	M01AC01
	214101BIJ	M01AC01
	214004ACH	M01AC01
	214002BIJ	M01AC01
	214001ACH	M01AC01
	214001ATB	M01AC01
	214004ATB	M01AC01
	214102BIJ	M01AC01
	214130BIJ	M01AC01
	214031BIJ	M01AC01
tenoxicam	235302ATB	M01AC02
	235301ATB	M01AC02
	235302BIJ	M01AC02
lornoxicam	355503ATB	M01AC05
	355501ATB	M01AC05
	355501ATB	M01AC05

	355503ATB	M01AC05
meloxicam	189701ACH	M01AC06
	189702ACH	M01AC06
	189701ACH	M01AC06
	189701ATD	M01AC06
	189702ATD	M01AC06
	189702ACH	M01AC06
	189701ATB	M01AC06
Propionic acid derivatives	186101ATB	M01AE
	249901ATB	M01AE
	493401ATB	M01AE
	216503ASY	M01AE
	249901ATB	M01AE
	493402ATR	M01AE
	186102ATR	M01AE
	186101BIJ	M01AE
	216504ACH	M01AE
	216534ASY	M01AE
	216501ASY	M01AE
ibuprofen	172806ATB	M01AE01
	172802ACS	M01AE01
	172811ASY	M01AE01
	172806ATB	M01AE01
	172802ATB	M01AE01
	172807ATB	M01AE01
	172840ASY	M01AE01
	172802ATB	M01AE01
	172804ASY	M01AE01
	172836ASY	M01AE01
	172806ACS	M01AE01
	172801ATB	M01AE01
	172813ASY	M01AE01
	172837ASY	M01AE01
	172834ASY	M01AE01
	172802ACH	M01AE01
	172806BIJ	M01AE01

	601402ATB	M01AE01
	172830BIJ	M01AE01
	172833ASY	M01AE01
	172807ATR	M01AE01
	172835ASY	M01AE01
	172812BIJ	M01AE01
	172802ACS	M01AE01
	172838ASY	M01AE01
	A38802ATB	M01AE01
	172832ASY	M01AE01
	D79701ATB	M01AE01
	172802ACH	M01AE01
naproxen	199501ATB	M01AE02
	199501ATB	M01AE02
	199402ACS	M01AE02
	199502ATB	M01AE02
	199404ATB	M01AE02
	199402ATB	M01AE02
	199501BIJ	M01AE02
	199501ACH	M01AE02
	199402ACH	M01AE02
	199404ATB	M01AE02
	199401ATR	M01AE02
	199407ASS	M01AE02
	199406ATB	M01AE02
	199504ATB	M01AE02
ketoprofen	179701BIJ	M01AE03
	179704BIJ	M01AE03
	179703ACH	M01AE03
	179731BIJ	M01AE03
	179731BIJ	M01AE03
	179804ACH	M01AE03
	179701ACR	M01AE03
	179704ACH	M01AE03
fenoprofen	157801ACH	M01AE04
	157803ATB	M01AE04

flurbiprofen	161902ATB	M01AE09
	161903ATB	M01AE09
tiaprofenic acid	238902ATB	M01AE11
	238902ACR	M01AE11
	238901ATB	M01AE11
	238901ATB	M01AE11
	245201BIJ	M01AE11
	238902ATR	M01AE11
oxaprozin	206101ATB	M01AE12
dexibuprofen	142301ATB	M01AE14
	142301ACS	M01AE14
	142303ATB	M01AE14
	142301ACS	M01AE14
	142303ATB	M01AE14
	142334ASY	M01AE14
	142302ATB	M01AE14
	142301ATR	M01AE14
	142301ACH	M01AE14
	142331ASY	M01AE14
	142304ASY	M01AE14
	142305ASY	M01AE14
	142302ATB	M01AE14
	142336ASY	M01AE14
	142337ASY	M01AE14
	142301ATB	M01AE14
	142332ASY	M01AE14
	142333ASY	M01AE14
dexketoprofen	454301ATB	M01AE17
	454301ATB	M01AE17
	454302BIJ	M01AE17
	454330BIJ	M01AE17
	454303BIJ	M01AE17
ibuprofen, combinations	D51001ATB	M01AE51
	D21900ACS	M01AE51
	384200ATB	M01AE51
	D52700ACS	M01AE51

	D51002ATB	M01AE51
	456600ASY	M01AE51
	441500AGN	M01AE51
	440900ASY	M01AE51
	D78300ACS	M01AE51
	D02400ATB	M01AE51
	D35400ACS	M01AE51
	A20000ATB	M01AE51
	D20600ATB	M01AE51
	C93600ATB	M01AE51
	D82500ATB	M01AE51
	D07100ATB	M01AE51
	A56900ATB	M01AE51
	D35900ATB	M01AE51
	C92900ACS	M01AE51
	D08100ACS	M01AE51
	441500ATB	M01AE51
	D20400ATB	M01AE51
	D03700ATB	M01AE51
naproxen and esomeprazole	523500ATB	M01AE52
	518000ATB	M01AE52
	527400ATB	M01AE52
mefenamic acid	189001ACH	M01AG01
	189001ACH	M01AG01
	189003ATB	M01AG01
	189001ATB	M01AG01
	189003ATB	M01AG01
	189001ACS	M01AG01
flufenamic acid	160301ACH	M01AG03
acetylsalicylic acid	110702ATB	N02BA01
	110702ATB	N02BA01
	110701ATB	N02BA01
	110802ATB	N02BA01
	110801ATB	N02BA01
	110902BIJ	N02BA01
	110802ATB	N02BA01

	110704ATB	N02BA01
	110706ATB	N02BA01
	110902BIJ	N02BA01
salsalate	225902ATB	N02BA06
diflunisal	460401ATB	N02BA11
imidazole salicylate	173502ATB	N02BA16
	173501ATB	N02BA16
	173502ATB	N02BA16
paracetamol	101401ATB	N02BE01
	101303ATB	N02BE01
	101404ATB	N02BE01
	101430ATR	N02BE01
	101332ASY	N02BE01
	101401ATB	N02BE01
	101305ATB	N02BE01
	101305ATB	N02BE01
	101404ATB	N02BE01
	101430ATR	N02BE01
	101330ASS	N02BE01
	101305ACH	N02BE01
	101306ATB	N02BE01
	101404ATR	N02BE01
	101303ATB	N02BE01
	101406ATB	N02BE01
	101403ASS	N02BE01
	101406ATB	N02BE01
	101405ATR	N02BE01
	101309BIJ	N02BE01
	101307ATB	N02BE01
	101433ASS	N02BE01
	101305ACS	N02BE01
	101309ATB	N02BE01
propacetamol	219401BIJ	N02BE05
	219401BIJ	N02BE05
	378000ATB	N02BE51
	A40000ATB	N02BE51

	383800ATB	N02BE51
	378100ATB	N02BE51
	C75000ATB	N02BE51
	A56100AGN	N02BE51
	252500ATB	N02BE51
	A57400ATB	N02BE51
	255400ATB	N02BE51
	384500APD	N02BE51
	384900ATB	N02BE51
	384600APD	N02BE51
	C51000ATB	N02BE51
	C75100ATB	N02BE51
	A82600AGN	N02BE51
	A52700ATB	N02BE51
	C89900ASY	N02BE51
	B02100ATB	N02BE51
	A75100ATB	N02BE51
	C56400ASY	N02BE51
	384000ATB	N02BE51
	330800CSP	N02BE51
	A75500ATB	N02BE51
	253800ATB	N02BE51
	A20200ATB	N02BE51
	A03200ATB	N02BE51
	A18900ATB	N02BE51
	D17400ACS	N02BE51
	A46700ATB	N02BE51
	384400APD	N02BE51
	384500ATB	N02BE51
	A31500ASY	N02BE51
	A04600ATB	N02BE51
	A43200ALQ	N02BE51
	C89800ASY	N02BE51
	D48000ATB	N02BE51
	384600ATB	N02BE51
	A75000ATB	N02BE51

	A28600APD	N02BE51
	A50000ATB	N02BE51
	A16700APD	N02BE51
	A59100ATB	N02BE51
	253500ATB	N02BE51
	384300APD	N02BE51
	B10800ATB	N02BE51
	A66400ATB	N02BE51
	A04200ATB	N02BE51
	A23700ATB	N02BE51
	B07800ATB	N02BE51
	E11500ACS	N02BE51
	A66700ATB	N02BE51
	A40300ATB	N02BE51
	D13600ACS	N02BE51
	D49100ATB	N02BE51
	A16800APD	N02BE51
	A61700ATB	N02BE51
	D17600ACS	N02BE51
	D56200ACH	N02BE51
	A23800ATB	N02BE51
	252000ATB	N02BE51
	A43500ALQ	N02BE51
	A46800ATB	N02BE51
	A04600ACS	N02BE51
	D01900ATB	N02BE51
	A28500APD	N02BE51
	A12300ATB	N02BE51
	252300ATB	N02BE51
	A43300ALQ	N02BE51
	A04500ATB	N02BE51
paracetamol combinations	C32900ATB	N02BE71

Supplementary Table 2. ATC codes of cough suppressants or other cold preparations

ATC name	Drug code	ATC code
codeine	137703ATB	R05DA04
noscapine	D12200ACH	R05DA07
dextromethorphan	142601ATB	R05DA09
combinations	254000ATB	R05DA20
	266600ASY	R05DA20
	A94000ACH	R05DA20
	266200ASY	R05DA20
	D99700ACH	R05DA20
	462300ACH	R05DA20
	B88200ACH	R05DA20
	560200ATB	R05DA20
	A63100ATB	R05DA20
	B88100ACH	R05DA20
	B87600ACH	R05DA20
	451100ATB	R05DA20
	568700ALQ	R05DA20
	B18200AGN	R05DA20
	A53400ATB	R05DA20
	A94200ASY	R05DA20
Other cough suppressants	A51503ATB	R05DB
benzonatate	115801ACS	R05DB01
benproperine	115401ATB	R05DB02
pentoxyverine	123601ATB	R05DB05
oxolamine	206601ATB	R05DB07
	206602ASY	R05DB07
butamirate	120701ASY	R05DB13
zipeprol	250402ASY	R05DB15
	250401ATB	R05DB15
dropropizine	150301ACH	R05DB19
combinations	E02100ACH	R05DB20
	C04800ACH	R05DB20
cloperastine	429001ASS	R05DB21
	429030ASS	R05DB21
	429031ASS	R05DB21
tipepidine	240001ATB	R05DB24

levodropropizine	183102ATB	R05DB27
	183135ASY	R05DB27
	183102ACH	R05DB27
	183101ASY	R05DB27
	183102ATB	R05DB27
	183103ATR	R05DB27
	183136AGN	R05DB27
	183134ASY	R05DB27
	183131ASY	R05DB27
COUGH SUPPRESSANTS AND EXPECTORANTS, COMBINATIONS	C57000AXS	R05F
	C04900ATT	R05F
	C15700ASY	R05F
	D27800AGN	R05F
	B40600ASY	R05F
	B30100ASY	R05F
Opium derivatives and expectorants	C57700ACH	R05FA
	428500ACH	R05FA
	268000ATB	R05FA
	532700ASY	R05FA
	B91300ACH	R05FA
	268600ACH	R05FA
	A53100ATB	R05FA
	567600ATB	R05FA
	433200ACH	R05FA
	265900ACH	R05FA
	A86000ACS	R05FA
	D68000ACS	R05FA
	D84200ATB	R05FA
	397000ACH	R05FA
	A86700ACH	R05FA
	D28700ACS	R05FA
	A95500ASY	R05FA
	266700ACH	R05FA
	C99000ACS	R05FA
	D01100ACS	R05FA
	D10700ACS	R05FA

	462400ACH	R05FA
	D22500ACS	R05FA
	C79800ACH	R05FA
	269000AGN	R05FA
	269100ASY	R05FA
	B20500ATB	R05FA
	485800ACS	R05FA
	A93800ACH	R05FA
	D29600ATB	R05FA
	267200ASY	R05FA
	456000ATB	R05FA
	A86100ACS	R05FA
	B08600ACH	R05FA
	C90100ACS	R05FA
	A62900ASY	R05FA
	A53200ATB	R05FA
	B52700ACH	R05FA
	268000ATB	R05FA
	416700ACH	R05FA
	A53500ASY	R05FA
	532800ASY	R05FA
opium derivatives and mucolytics	B05700ACH	R05FA01
	A86200ACH	R05FA01
	485800ACH	R05FA01
	D99800ACH	R05FA01
	D99600ACH	R05FA01
opium derivatives and expectorants	E01300ACH	R05FA02
	B66700AGN	R05FA02
	C11900AGN	R05FA02
	D42000ACS	R05FA02
	532500ASY	R05FA02
	532600ASY	R05FA02
	419600ACH	R05FA02
	D98100ACH	R05FA02
	C90000ACS	R05FA02
	B89400ASY	R05FA02

	B49700ASY	R05FA02
	267600ASY	R05FA02
	266500ATB	R05FA02
	266300ASY	R05FA02
	A73000ASY	R05FA02
	B44400ASY	R05FA02
	B09400ASY	R05FA02
	A62700ACS	R05FA02
	B94700AGN	R05FA02
	A62800ACS	R05FA02
	A78700ACS	R05FA02
	C13800ACH	R05FA02
	B85800ASY	R05FA02
	A62700ACH	R05FA02
	557700ASY	R05FA02
	442500ACS	R05FA02
	D34800ATB	R05FA02
	E00800ACH	R05FA02
	458200ACH	R05FA02
	D55700ASY	R05FA02
	B47900ACH	R05FA02
	A86000ACH	R05FA02
	A38300ACS	R05FA02
	D45100ACS	R05FA02
	B20400ASY	R05FA02
	B28200ACH	R05FA02
	B21200ATB	R05FA02
	B40400AGN	R05FA02
	D51500ACS	R05FA02
	E10800ACS	R05FA02
	B36300ACH	R05FA02
	C94300ACS	R05FA02
	266500ATB	R05FA02
	E01700ACH	R05FA02
	B43200ACH	R05FA02
	255600ACH	R05FA02

	A86800ACH	R05FA02
	D55600ATB	R05FA02
	267300ASY	R05FA02
	C65900ACH	R05FA02
	C74900ACH	R05FA02
	C86100ACS	R05FA02
	A86300ACH	R05FA02
	395900ACS	R05FA02
	D58600ACS	R05FA02
	A68800ACS	R05FA02
	D45000ACS	R05FA02
	A86700ACH	R05FA02
	C93300ACS	R05FA02
	458300ACH	R05FA02
Other cough suppressants and expectorants	E02700ACH	R05FB
	B43300ASY	R05FB
	396500ASY	R05FB
	B58400ACE	R05FB
	B35400ATB	R05FB
	335100CSP	R05FB
	C56200ATB	R05FB
	581000ASY	R05FB
	B52500ACH	R05FB
	B47700ACH	R05FB
	266800ASY	R05FB
	B20700APD	R05FB
	335000CSP	R05FB
cough suppressants and mucolytics	B25800ATT	R05FB01
	396600ASY	R05FB01
cough suppressants and expectorants	D14200ASY	R05FB02
	A97200ASY	R05FB02
	476100ASY	R05FB02
	B20000ACH	R05FB02
	E01800ACH	R05FB02
	E00400ACH	R05FB02
	E03200ACH	R05FB02

	D12300ASY	R05FB02
	C94900ASY	R05FB02
	B40300ACH	R05FB02
	B94800ALQ	R05FB02
	C24100ACH	R05FB02
	B16300ACH	R05FB02
	A94600ALQ	R05FB02
	B31000ACH	R05FB02
	B37400ACH	R05FB02
	A29100ACH	R05FB02
OTHER COLD PREPARATIONS	A16700AGN	R05X
	B18000ATB	R05X
	A56200AGN	R05X
	C24200ACH	R05X
	A88500AGN	R05X
	A89000ACH	R05X
	C08100ACH	R05X
	B95500ACH	R05X
	B05300ACH	R05X
	C08200ACH	R05X
	D98400ACH	R05X
	C98100ACH	R05X
	D29500ACH	R05X
	D33200ACH	R05X
	E00300ACH	R05X
	D51400ALQ	R05X
	D73800ACH	R05X
	A06500ATB	R05X
	A57800ATB	R05X
	A18900ATB	R05X
	253400ATB	R05X
	B30700ACE	R05X
	252700ATB	R05X
	A28100APD	R05X
	A80500ASY	R05X
	D94000ACH	R05X

	D50200ACH	R05X
	A49900ACH	R05X
	A53800ATB	R05X
	A06400ATB	R05X
	D12500ATB	R05X
	A03900ATB	R05X
	433100ATB	R05X
	E03400ATB	R05X
	E01400ATB	R05X
	B07100ATB	R05X
	B40900ATB	R05X
	E03600ATB	R05X
	B41000ATB	R05X
	A18700ACS	R05X
	D27900ACS	R05X
	D28000ACS	R05X
	D28100ACS	R05X
	D28200ACS	R05X
	A39200ATB	R05X
	A05500ATE	R05X
	A19700ATB	R05X
	A79600ACH	R05X
	D11200ATB	R05X
	255200ACH	R05X
	B00100ACH	R05X
	B96600ATB	R05X
	A79300ATB	R05X
	B18400ATB	R05X
	D14700ATB	R05X
	C08300ACH	R05X
	A52500ACH	R05X
	D96900ACH	R05X
	D39500ATB	R05X
	A99900ACH	R05X
	A79000ACH	R05X
	367100ASY	R05X

	D84500ATB	R05X
	254000ATB	R05X
	A61800ATE	R05X
	254900ACH	R05X
	A53400ATB	R05X
	C13200ACH	R05X
	B09700ACH	R05X
	A55500ACS	R05X
	A19800ATB	R05X
	A39000ACS	R05X
	C78100ACH	R05X
	A86500ACS	R05X
	D03800ACS	R05X
	D37800ACH	R05X
	A28100ATB	R05X
	A53900ATB	R05X
	A50100ACS	R05X
	A78900ACS	R05X
	E03700ACH	R05X
	D29300AGN	R05X
	E03500ACH	R05X
	D55100ACH	R05X
	252700ACS	R05X
	A57500ACH	R05X
	571600ACS	R05X
	A57700ACH	R05X
	467900ACS	R05X
	A53300ACS	R05X
	A19000ACS	R05X
	A07000ACS	R05X
	A63000ACS	R05X
	A81600ACS	R05X
	A81700ACS	R05X
	A63200ACS	R05X
	C89700ACS	R05X
	C90200ACS	R05X

	C89600ACS	R05X
	A25000ACS	R05X
	C92600ACS	R05X
	C92500ACS	R05X
	C92400ACS	R05X
	C92300ACS	R05X
	C92200ACS	R05X
	C94200ACS	R05X
	C93700ACS	R05X
	C95600ACS	R05X
	C95700ACS	R05X
	C95500ACS	R05X
	C95400ACS	R05X
	C97300ACS	R05X
	C97600ACS	R05X
	C99100ACS	R05X
	C96200ACS	R05X
	C98900ACS	R05X
	D01600ACS	R05X
	D00800ACS	R05X
	C99200ACS	R05X
	D10800ACS	R05X
	D11800ACS	R05X
	D13500ACS	R05X
	D13800ACS	R05X
	D15800ACS	R05X
	D17200ACS	R05X
	D17500ACS	R05X
	D17300ACS	R05X
	D19700ACS	R05X
	D19500ACS	R05X
	D22200ACS	R05X
	D22300ACS	R05X
	D22400ACS	R05X
	D22100ACS	R05X
	D22000ACS	R05X

	D27100ACS	R05X
	D84900ACS	R05X
	D37400ACS	R05X
	D37500ACS	R05X
	D37600ACS	R05X
	D37700ACS	R05X
	D40300ACS	R05X
	D41100ACS	R05X
	D94900ACS	R05X
	D44500ACS	R05X
	D57000ACS	R05X
	D61100ACS	R05X
	D61200ACS	R05X
	D74600ACS	R05X
	D79800ACS	R05X
	D62000ACS	R05X
	E12500ACS	R05X
	E12100ACS	R05X
	E12800ACS	R05X
	C19400ACH	R05X
	D60400ACS	R05X
	A50800ACH	R05X
	D10900ACS	R05X
	433000ATB	R05X
	C04700ACH	R05X
	B88900ACH	R05X
	B19400ACH	R05X
	A91200ALQ	R05X
	B88400ACH	R05X
	D08400ACS	R05X
	D36600ASY	R05X
	D36700ASY	R05X
	D41900ACS	R05X
	C59600ACS	R05X
	D41800ACS	R05X
	D81900ATB	R05X

	E00400ACH	R05X
	378000ATB	R05X
	A63900ATB	R05X
	B10900ACH	R05X
	A25400ATB	R05X
	A30300ATB	R05X
	C41800ALQ	R05X
	B05100ACH	R05X
	D82200ACH	R05X
	E01800ACH	R05X
	B08500ACH	R05X
	B51100ACH	R05X
	B08400ALQ	R05X
	C34400ALQ	R05X
	A65600ALQ	R05X
	A98300ACH	R05X
	B98600ACH	R05X
	458300ACH	R05X
	A52500ATB	R05X
	D82000ATB	R05X
	E00700ACH	R05X
	B89000ACE	R05X
	255300ACH	R05X
	A25000ACH	R05X
	D14800ATB	R05X
	D40600ATB	R05X
	D75800ATB	R05X
	B05900ACH	R05X
	A56000ASY	R05X
	265500ASY	R05X
	A79100ACH	R05X
	C43400ACH	R05X
	A55900ASY	R05X
	B87300ASY	R05X
	B90200ACH	R05X
	A51100ATB	R05X

	D11900ACS	R05X
	D44100ASY	R05X
	D46300ASY	R05X
	D46400ASY	R05X
	D63100ACS	R05X
	D82400ACH	R05X
	B30400AGN	R05X
	D61400ALQ	R05X
	D07600ALQ	R05X
	B96700ACH	R05X
	D97800ACH	R05X
	A51200ACH	R05X
	A50900ACH	R05X
	D52300ATB	R05X
	D54700ATB	R05X
	D54300ATB	R05X
	D60900ALQ	R05X
	D61000ALQ	R05X
	D54800ASY	R05X
	B98500AGN	R05X
	A29400ACH	R05X
	255100ACE	R05X
	B09500ASY	R05X
	B89100ACE	R05X
	C01800ASY	R05X
	A79200ASY	R05X
	B13900ASY	R05X
	D97200ACH	R05X
	B86300ASY	R05X
	D94100ACH	R05X
	A99700ACE	R05X
	B40200ACH	R05X
	C01200ACH	R05X
	D19100ACH	R05X
	D82300ACH	R05X
	D99400ATB	R05X

	A18400ATB	R05X
	B86600ALQ	R05X
	C94700ACS	R05X
	D53600ALQ	R05X
	D53200ALQ	R05X
	D53900ALQ	R05X
	E06500ACS	R05X
	456000ATB	R05X
	D65500ACS	R05X
	D65200ACS	R05X
	B28500ACH	R05X
	D12600ATB	R05X
	D54400ASY	R05X
	D66700ASY	R05X
	D66400ASY	R05X
	D66800ASY	R05X
	C55400AGN	R05X
	A79400ATB	R05X
	D14400ATB	R05X
	E00500ACH	R05X
	B98900ATB	R05X
	D12200ACH	R05X
	A82100ATB	R05X
	B17500ACH	R05X
	C89500ACH	R05X
	D10500ACH	R05X
	E01900ACH	R05X
	E14000ACH	R05X
	C36900ALQ	R05X
	A78900ALQ	R05X
	A04400ATB	R05X
	B86200ASY	R05X
	B10200ALQ	R05X
	C13300ACH	R05X
	C93800ACS	R05X
	E01500ACH	R05X

	D76100ACS	R05X
	D76400ACS	R05X
	D76200ACS	R05X
	E08100ATB	R05X
	C21300AGN	R05X
	D87500ATB	R05X
	B10100ALQ	R05X
	C36800ALQ	R05X
	B94800ALQ	R05X
	B87700ACH	R05X
	B20600ALQ	R05X
	B58400ACE	R05X
	D03000ATB	R05X
	D03100ATB	R05X
	D02800ATB	R05X
	D02900ATB	R05X
	D08500ALQ	R05X
	D21200ACH	R05X
	D21100ACH	R05X
	E09200ACS	R05X
	E10100ACS	R05X
	B54200ACH	R05X
	A93400ASY	R05X
	D24500ACS	R05X
	E01600ACH	R05X
	D97700ACH	R05X
	D47000ASY	R05X
	D66300ALQ	R05X
	266900ACH	R05X
	D83900ALQ	R05X
	B24300APD	R05X
	A96500APD	R05X
	B54100ASY	R05X
	A23400ATB	R05X
	A64900ATB	R05X
	A72100ATB	R05X

	D98900ALQ	R05X
	B17100ALQ	R05X
	B88300ACH	R05X
	254500ASY	R05X
	B05200ALQ	R05X
	B20200ACH	R05X
	B87000ACH	R05X
	D97600ATB	R05X
	B18700ACH	R05X
	D13700ACS	R05X
	E00600ACH	R05X
	D68300ACH	R05X
	D69000ACH	R05X
	C55700ACE	R05X
	C96500ACH	R05X
	D03500ACH	R05X
	D10100ACH	R05X
	E02800ATB	R05X
	B18400ACH	R05X
	D48900ACH	R05X
	D44400ATB	R05X
	D70600ACS	R05X
	D71300ACS	R05X
	D71400ACS	R05X
	D03900ASY	R05X
	D43700ACS	R05X
	D43800ACS	R05X
	B06000ACS	R05X
	C53800ATB	R05X
	B89100ACH	R05X
	C18300ACH	R05X
	573600AGN	R05X
	577200AGN	R05X
	D87700ATB	R05X
	D94200ACH	R05X
	A79800ACH	R05X

	A87900ACH	R05X
	A86900ACH	R05X
	255000ACH	R05X
	A84400ACH	R05X
	A58800ACH	R05X
	A66500ACH	R05X
	D73300ATB	R05X
	B58500ACH	R05X
	D98200ACH	R05X
	D97300ACH	R05X
	C93900ATB	R05X
	C93900ACH	R05X
	D93900ACH	R05X
	D47300AGN	R05X
	A90500ATB	R05X
	A62500ATB	R05X
	D22600ACS	R05X
	D23400ACH	R05X
	D62100ACS	R05X
	C36700ALQ	R05X
	B09900ACH	R05X
	C15600ASY	R05X
	D87600ATE	R05X
	A79700ATB	R05X
	D95400ALQ	R05X
	D84700ATB	R05X
	D92400ASY	R05X
	B06100ATB	R05X
	C79000ASY	R05X
	A78600ACH	R05X
	B52600ACH	R05X
	B47600ASY	R05X
	A78800ATB	R05X
	B08200ALQ	R05X
	B08300ALQ	R05X
	B36600ACH	R05X

	E05600ACH	R05X
	D68100ACS	R05X
	D77400ACS	R05X
	E04700ACS	R05X
	E04800ACS	R05X
	E11900ACS	R05X
	E12400ACS	R05X
	E12700ACS	R05X
	D28300ACH	R05X
	D43500ACS	R05X
	B06200ACH	R05X
	D55500ASY	R05X
	D58400ASY	R05X
	D58500ASY	R05X
	D32100ACH	R05X
	A99100ACH	R05X
	C24500ACH	R05X
	A86500ACH	R05X
	447400ACS	R05X
	E01200ACH	R05X
	D21400AGN	R05X
	D48100ACH	R05X
	D49500ACH	R05X
	D49300ACH	R05X
	D88000ACH	R05X
	D94300ACH	R05X
	D54600ACH	R05X
	D84600ACH	R05X
	D08400ACH	R05X
	C15800ASY	R05X
	B86100ASY	R05X
	D12000ACH	R05X
	D13900ACH	R05X
	D63200ASY	R05X
	D23800ACS	R05X
	D24000ACS	R05X

	D23900ACS	R05X
	D27200ACS	R05X
	D28500ACS	R05X
	D28800ACS	R05X
	D91500ACS	R05X
	D85000ACS	R05X
	D44600ACS	R05X
	D44700ACS	R05X
	D46600ACS	R05X
	D46700ACS	R05X
	D87900ACS	R05X
	D51700ACS	R05X
	D97900ACS	R05X
	D97000ACS	R05X
	D60000ACS	R05X
	D59100ACS	R05X
	D60300ACS	R05X
	D94400ACS	R05X
	D57500ACS	R05X
	E04200ACS	R05X
	E11000ACS	R05X
	E12000ACS	R05X
	E11700ACS	R05X
	E11800ACS	R05X
	E12600ACS	R05X
	E12300ACS	R05X
	D53800ASY	R05X
	B34100ACH	R05X
	B04000ATB	R05X
	B37900ACH	R05X

Supplementary Table 3. Distribution of sentinel sites for influenza-like illness surveillance system in Korea

	Number of sentinel sites (2014-2016)	2018-2019
Total	200	199
Seoul	38	37
Busan	13	12
Daegu	10	10
Incheon	12	11
Gwangju	6	7
Daejeon	6	6
Ulsan	4	6
Gyeonggi	47	47
Gangwon	6	6
Chungbuk	6	6
Chungnam	8	6
Jeonbuk	8	8
Jeonnam	8	8
Gyeonbuk	10	10
Gyeongnam	14	14
Jeju	4	5

Supplementary Figure 1. Epidemic description from the moving epidemic methods for the 2014-2018 ILI rates: The KCDC sentinel data vs. National Health Insurance claims data

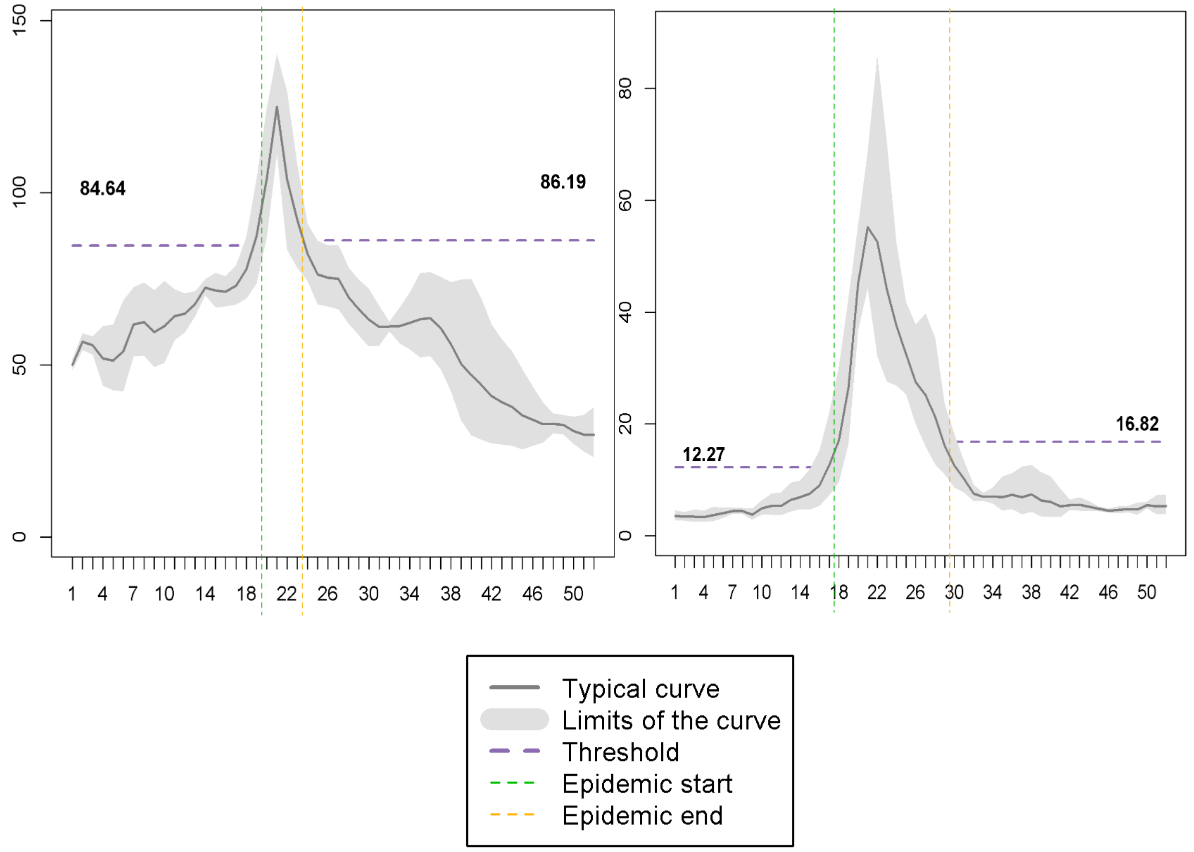

Supplementary Figure 2. The nationwide Drug Utilization Review (DUR) system process in Korea (adapted from the Health Insurance Review and Assessment Service)

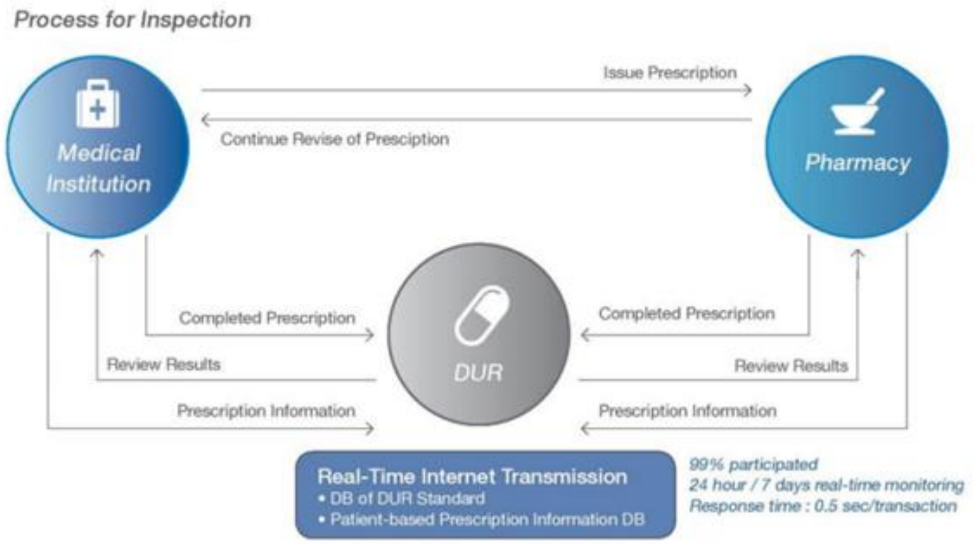

Supplement: Supplementary file 1 — Supplementary Information. [file 41598_2021_90511_MOESM1_ESM.pdf]
